# POSITIVE ASPECTS OF DEMENTIA CAREGIVING EVOLVE FROM AN INTEGRATIVE STRESS COPING AND EXISTENTIALISM PARADIGMS

**DOI:** 10.1093/geroni/igad104.0144

**Published:** 2023-12-21

**Authors:** Doris S F Yu, Sheung Tak Cheng

**Affiliations:** The University of Hong Kong, Hong Kong, Hong Kong; The Education University of Hong Kong, Hong Kong, Hong Kong

## Abstract

Family caregiving is the crucial informal care resource to buffer the associated disease burden. Research in this field has focused on promoting effective adaptation of family caregivers of persons with dementia (PWD) by ameliorating their caregiving burden. Little attention is placed on positive aspects of caregiving (PAC). PAC refers to the extent to which caregiving experience is appraised as enriching to one’s life, and promoting uplift, personal growth and gratification. This mixed-method study investigated how PAC evolved from the encounter of the family caregivers of PWD. Based on the research evidence about how PAC is shaped, an integrated theoretical model combining the stress coping and existential paradigms were hypothesized to explain the evolvement of PAC. A mixed-method longitudinal study was conducted, and 403 family caregivers of PWD were invited to respond on validated measure for active dementia management strategies, self-efficacy, social support, meaning-making, motivation to care and religiosity. PAC was measured at baseline and 6 months thereafter. Path analysis indicated that PAC was evolved from the self-efficacy derived from active coping with the caregiving experience, with a social support as a significant moderator for such relationship. The use of meaning-focus coping especially in a religious context also optimize this favorable caregiving attribute. The qualitative findings converge with this model, and further elaborate how performance success, self-affirmation, spiritual growth, and relationship solidarity consolidate perceived positivity on the caregiving experience. This study provides important insights on how to advance caregiving supportive care by adding an existential paradigm.

